# CDG due to Defective Membrane Transporters: Update

**DOI:** 10.1002/jimd.70133

**Published:** 2026-01-19

**Authors:** D. Quelhas, C. R. Ferreira, J. Jaeken

**Affiliations:** ^1^ Unidade de Bioquímica Genética, Serviço de Genética Laboratorial, Centro de Genética Médica, Clínica de Genética e Patologia Centro Hospitalar Universitário de Santo António, Unidade Local de Saúde de Santo António Porto Portugal; ^2^ Unit for Multidisciplinary Research in Biomedicine ICBAS, UP Porto Portugal; ^3^ Centro Referência Doenças Hereditárias do Metabolismo, Centro Hospitalar Universitário de Santo António Unidade Local de Saúde de Santo António Porto Portugal; ^4^ Unit on Skeletal Genomics, Eunice Kennedy Shriver National Institute of Child Health and Human Development, National Institutes of Health Bethesda Maryland USA; ^5^ Center for Metabolic Diseases University Hospital Gasthuisberg, KU Leuven Leuven Belgium

## Abstract

Congenital disorders of glycosylation are genetic defects in the glycoprotein and glycolipid glycan assembly and attachment. Some 200 CDG have been reported since the first clinical description in 1980. Most CDG are enzymatic deficiencies, but 13 (6.5%) are defects in the ER, Golgi apparatus (GA), and plasma membrane transporters. This review provides an update on the clinical, biochemical, genetic, and therapeutic aspects of these disorders and on animal models. Defects in other cellular trafficking mechanisms have been excluded from this update.

## 1 Introduction

Glycosylation is the synthesis of fully functional glycans, and their covalent enzymatic attachment to other molecules including proteins, lipids, and small RNA [1]. CDG are human pathological conditions caused by genetic and de novo variants in any pathway that alters normal glycosylation [2]. In 2009, Jaeken and colleagues proposed a new nomenclature for CDG, using (only) the official gene symbol followed by “‐CDG.” It is widely used by the CDG community and has proven to be appropriate for the still growing number of disorders [3].

While glycosylation mainly occurs inside the endoplasmic reticulum (ER) and GA in eukaryotes, nucleotide sugars are produced in the cytoplasm and nucleus. Newly created nucleotide sugars must thus be carried into the ER and GA since they are on the “wrong” side of the membrane for most glycosylation processes. These donors cannot just diffuse into these compartments because of their negative charge. Nucleotide sugars are delivered into the lumen of these organelles by a set of energy‐independent nucleotide sugar antiporters in eukaryotic cells. At the same time, nucleoside monophosphates—the majority of which must first be produced from the nucleoside diphosphates by nucleoside diphosphatases—exit simultaneously [1].

**FIGURE 1:**
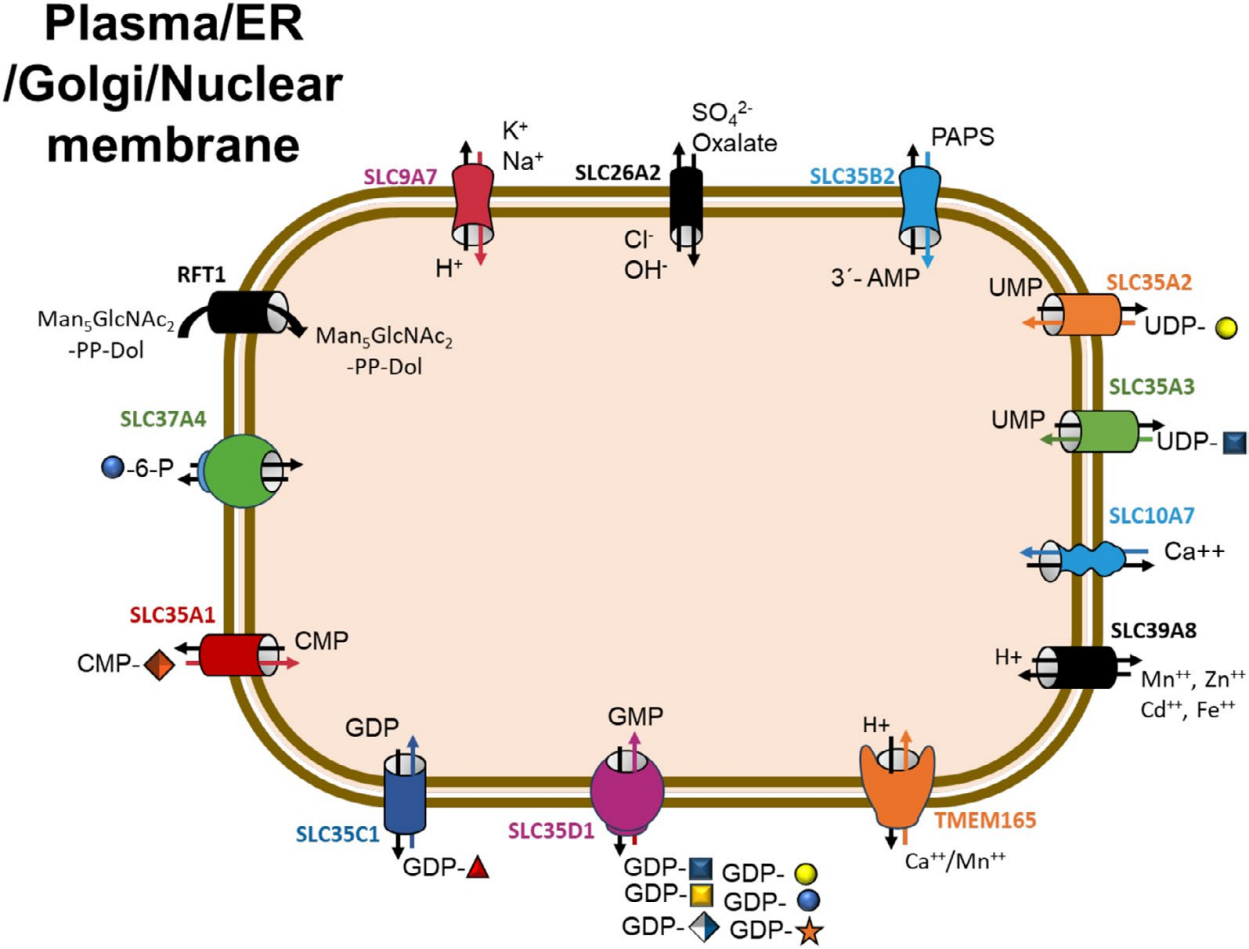
Schematic representation of transporters located not only in the GA membrane but also in the ER, plasma, and nuclear membrane, carrying either nucleotide sugars, PAPS, activated sugars, or ions. These nucleotide sugar proteins are antiporters and return the corresponding nucleoside monophosphate back to the cytoplasm when the nucleotide sugar is delivered into the ER or GA.

Mirroring the extensive range of compounds requiring transport, different types of transporter proteins have evolved that can be subdivided into four main superfamilies: (a) the ATP‐binding cassette (ABC) transporters [4, 5], (b) ATPases [6, 7], (c) ion channels [8], and (d) solute carriers (SLC) [9, 10]. Most CDG transporter defects are related to the SLC superfamily of transporter proteins (Figure 1). Members of this superfamily are found in the membrane of almost every organelle, including the ER and GA, as well as in the plasma membrane.

SLCs are integral membrane transporter proteins that control essential physiological functions, including nutrient uptake, ion transport and waste removal [10, 11]. The number of known human SLC genes is over 450. The root symbol SLC is followed by a numeral (e.g., SLC1, solute carrier family 1), followed by a letter which defines the subfamily (only A is used when the family has not been subdivided), and finally, a number designating the individual transporter gene (e.g., SLC3A1). Transporters are assigned to a specific family if the encoded protein has at least 20% amino acid sequence identity to other family members.

This review provides an update on the clinical, biochemical, genetic, and therapeutic aspects of CDG transporter defects and on animal models of these defects.

## 2 ER Transporter Defects

### 2.1 RFT1‐CDG

#### 2.1.1 Clinical Presentation

RFT1‐CDG was first reported in 2008 [12]. Currently, 18 patients are known belonging to 17 families (overview in [13, 14]). Common to all these patients is (mostly severe) psychomotor disability. The following symptoms were present in the majority of the patients: epilepsy (14), including burst‐suppression [15], hypotonia (13), sensorineural hearing loss (12), and dysmorphism (10). Nine patients showed feeding problems. A large number of (mostly neurological) symptoms have been reported in a minority of patients: failure to thrive, short length, arthrogryposis, ataxia, basal ganglia lesions, behavioral problems, cerebral and/or cerebellar atrophy, hyperreflexia, hypertonia, microcephaly, spastic tetraparesis, visual impairment, white matter abnormalities, convergent strabismus, glaucoma, pale optic nerves, respiratory problems, hepatomegaly, and deep venous thrombosis.

Five patients died between ages 24 days and 4.25 years (due to respiratory insufficiency, cardiorespiratory arrest or pulmonary embolus). The oldest patients (siblings) were 19 and 21 years at the time of publication [16].

#### 2.1.2 Metabolic Derangement and Genetics

RFT1‐CDG is caused by variants in *RFT1*. The gene product catalyzes the translocation of the oligosaccharide‐lipid intermediate Man5GlcNAc2‐PP‐Dol from the cytoplasm‐facing leaflet of the ER membrane to the ER's lumen leaflet. This flippase‐mediated process is ATP‐independent. According to some studies, this “flippase” would not be required for flipping this heptasaccharide lipid in microsomes or unilamellar vesicles reconstituted with ER membrane proteins [17]. However, recent data seem to confirm the molecular identity of Rft1 as the Man_5_GlcNAc_2_‐PP‐Dol ER flippase [18]. It is a multispanning membrane protein with N and C termini facing the cytoplasm. It is not *N‐*glycosylated. RFT1‐CDG has an autosomal recessive inheritance. So far, 14 variants have been reported; the majority map to highly conserved regions of the protein [17].

#### 2.1.3 Diagnostic Testing and Treatment

This CDG has to be considered mainly in the presence of psychomotor disability and hypotonia associated with sensorineural hearing loss and/or early‐onset severe epilepsy. The standard diagnostic test is serum transferrin (Tf) isoelectrofocusing (IEF) showing a type 1 pattern. However, this test can be normal as shown by Aeby et al. in 2016 in patient 2 (normal result at 4 days, at 6 months and at 2 years).

Treatment is only symptomatic.

### 2.2 SLC35D1‐CDG

#### 2.2.1 Clinical Presentation

In 1986, Borochowitz et al. described a lethal neonatal chondrodysplasia with a snail‐like pelvis on radiographic imaging and called it Schneckenbecken dysplasia [19]. In 2007, Hiraoka et al. identified pathogenic variants in *SLC35D1* as the cause of this syndrome [20]. This protein is expressed in the ER and is believed to be a general UDP‐sugar transporter [21]. Fifteen patients have been reported, belonging to 8 families (review in [22]). Nine of them showed a syndrome with prenatal or early neonatal death and short, thick, long bones, thoracic hypoplasia, severe flattening of the vertebral bodies, and a snail‐like pelvis.

Mild skeletal dysplasia was present in the other SLC35D1‐CDG patients, namely a family with two siblings, their two cousins and a paternal uncle aged 4 to 31 years [23] and a 17‐year‐old male [22]. They presented with relative macrocephaly, coarse face, short neck, narrow chest, short extremities, brachydactyly of hands and feet, genua valga, scoliosis, and absence of the characteristic snail‐like appearance of the ilia.

#### 2.2.2 Metabolic Derangement and Genetics

Recent work has shown evidence that SLC35D1 is not only a transporter of UDP‐GlcNAc, UDP‐GalNAc, and UDP‐GlcA but is also capable of transporting UDP‐Gal, UDP‐Glc, and UDP‐Xyl [21]. This ability broadens its role beyond the polymerization of chondroitin sulfate. Ten variants have been reported, including four missense, two nonsense, two deletion, and two splice variants.

#### 2.2.3 Diagnostic Testing and Treatment

The clinical presentation, at least the severe one, is pathognomonic and prompts genetic testing of SLC35D1. No treatment is available.

## 3 GA Transporter Defects

### 3.1 SLC9A7‐CDG

#### 3.1.1 Clinical Presentation

Six patients (from two unrelated families: one family with three affected brothers and a maternal nephew, and another with a boy and maternal uncle) have been reported with a convincing diagnosis of this x‐linked intellectual disability disorder without obvious dysmorphism [24]. Psychomotor and intellectual development was moderately to severely affected. Some patients showed central hypotonia and brisk tendon reflexes. The four patients in the first family reached adulthood.

#### 3.1.2 Metabolic Derangement and Genetics

These patients show a missense variant (c.1543C>T;p.Leu515Phe) in *SLC9A7* encoding an alkali cation/proton exchanger. This protein resides in the GA, particularly in the *trans‐*Golgi network and post‐Golgi vesicles, mediating the electroneutral influx of Na^+^ or K^+^ in exchange for H^+^. It may contribute to regulating the GA volume and pH [25].

#### 3.1.3 Diagnostic Testing and Treatment

In the one family that was tested, the capillary zone electrophoresis of serum Tf was normal, but mass spectrometry showed an increased trisialo‐oligo/disialo‐oligo ratio, suggesting an absence of a single sialic acid moiety (type 2 pattern).

No treatment is available.

### 3.2 SLC10A7‐CDG

#### 3.2.1 Clinical Presentation

SLC10A7‐CDG was first reported in 2018 [26, 27]. Thirteen patients belonging to 12 families have been published [28, 29]. They show a unique phenotype mainly characterized by skeletal dysplasia and *amelogenesis imperfecta*. The complete skeletal picture comprises pre‐ and postnatal short stature, short long bones, Pierre‐Robin sequence, cleft palate, micrognathia, abnormal vertebrae, chest deformity, hyperlordosis, kyphoscoliosis, dislocation of large joints, *genua valga*, “monkey wrench” appearance of proximal femora, advanced carpal/tarsal ossification, and small epiphyses. Hypomineralized *amelogenesis imperfecta* is the other constant feature. Most patients also suffer from some degree of hypoacusis. Less frequent features are mild to borderline intellectual disability, optic nerve atrophy, hypermetropia, myopia, astigmatism, short nose and cardiac defects. Ages at publication ranged from 6 months to 33 years.

#### 3.2.2 Metabolic Derangement and Genetics

SLC10A7 is a 10‐transmembrane transporter homodimer. Based on current knowledge, the location of SLC10A7 is predominantly in the GA and it affects Ca^2+^ homeostasis in the secretory pathway [30]. Other family members transport Na^+^/bile acids, but SLC10A7 is still an orphan solute carrier. Patients have an N‐glycosylation defect comprising increased high‐mannose glycans, glycans lacking GlcNAc, and decreased sialylated glycans. Calcium labeling of a patient's fibroblasts showed that Ca^++^ is primarily retained in the nucleus, while in control cells it is mainly in the cytoplasm. This retention suggests that SLC10A7 regulates calcium trafficking and is possibly a calcium transporter. Genetic analysis showed nine variants and a complete loss of *SLC10A7* mRNA in two siblings [28].

#### 3.2.3 Diagnostic Testing and Treatment

*SLC10A7* sequencing is indicated in patients with the association of skeletal dysplasia and *amelogenesis imperfecta*, particularly in the presence of hypoacusis.

Treatment mainly consists of surgical management of the spine.

### 3.3 SLC26A2‐CDG

#### 3.3.1 Clinical Presentation

This is a defect in the sulfate transporter [31, 32, 33].

Pathogenic variants in *SLC26A2* lead to a wide spectrum of both lethal and non‐lethal skeletal dysplasia. The lethal conditions include achondrogenesis type 1B and atelosteogenesis type II. The non‐lethal conditions are diastrophic dysplasia (DTD) and recessive multiple epiphyseal dysplasia (rMED).

Achondrogenesis type 1B phenotype is characterized by deficient ossification of the lumbar vertebrae and absent ossification of the sacral, pubic, and ischial bones. In addition to severe micromelia, there is a disproportionately large cranium due to marked oedema of soft tissues. Another phenotype is atelosteogenesis type II, a skeletal syndrome including shortening of the limbs. Patients from both dysplasias are stillborn or die soon after birth.

DTD is found in almost all populations but is exceptionally common in the Finnish population. In 1990, about 160 subjects with DTD were known in Finland. Over 250 subjects with DTD have been published elsewhere. Clinical features of DTD include short stature with limb shortening, contractures of large joints, spinal deformities, cleft palate, clubfoot, cystic swelling of the external ear and deformities of the hands. Clinical findings of the milder phenotype, rMED, have normal/mildly shortened stature, joint contractures, mild hand deformity, double‐layered patellae and clubfoot. The clinical features partly overlap with those of DTD [34].

#### 3.3.2 Metabolic Derangement and Genetics

SLC26A2 is a sulfate transporter important for synthesizing sulfated proteoglycans in cartilage. This protein is essential for chondrocyte proliferation, differentiation, and cell size expansion [35]. It mediates electroneutral anion exchange of sulfate ions for oxalate ions and sulfate and oxalate ions for chloride ions. The coupling of sulfate transport to both hydroxyl and chloride ions likely serves to ensure transport on the one hand at acidic pH when most sulfate uptake is mediated by sulfate‐hydroxide exchange and on the other hand at alkaline pH when most sulfate uptake is mediated by sulfate‐chloride exchange [36, 37].

Most Finnish patients are homozygous for the founder variant c.‐26+2T>C. The Finnish DTD founder variant is characterized by a GT‐to‐GC transition at the splice donor site of a previously uncharacterized 5′ untranslated exon of the SLC26A2 gene. This variant significantly reduces *SLC26A2* mRNA levels and is prevalent in the Finnish population, with about 90% of DTD chromosomes carrying this splice site variant. In a study of 84 Finnish families affected by DTD, 69 families had patients homozygous for this variant, 14 were heterozygous, and one family had none. The most common variant outside of Finland is p.Arg279Trp, which leads to a milder rMED phenotype in homozygosity.

#### 3.3.3 Diagnostic Testing and Treatment

*SLC26A2* sequencing is indicated in patients with the above‐mentioned skeletal dysplasias. No treatment is available, although N‐acetylcysteine has been proposed as a sulfate donor and showed promise in preclinical models [38, 39].

### 3.4 SLC35A1‐CDG

#### 3.4.1 Clinical Presentation

Only four patients, including two siblings, have been reported with this CMP‐sialic acid transporter defect [40, 41, 42]. They showed a neurological syndrome, and three of them also had macrothrombocytopenia with a bleeding diathesis. The neurological picture consisted of developmental/intellectual disability, ataxia, epilepsy, and one to three behavior problems, choreiform movements, microcephaly, and hypotonia.

#### 3.4.2 Metabolic Derangement and Genetics

SLC35A1 can mediate the transport not just of CMP‐sialic acid, but also of other nucleotide‐sugars, such as CDP‐ribitol [43]. Laboratory investigation shows giant platelets, thrombocytopenia, a type 2 serum Tf IEF pattern, platelet hyposialylation, and decreased N‐ and O‐glycans' sialylation. One patient also showed proteinuria and aminoaciduria [41]. Two homozygous and two heterozygous missense variants were found.

#### 3.4.3 Diagnostic Testing and Treatment

In patients with encephalopathy and macrothrombocytopenia, serum Tf IEF is recommended. Finding a type 2 pattern should prompt an in vitro test of CMP‐sialic transport. Treatment is limited to fresh platelet transfusions.

### 3.5 SLC35A2‐CDG

#### 3.5.1 Clinical Presentation

This CDG was first described by Ng et al. [44] and Kodera et al. in 2013 [45]. Some 80 individuals have been reported with this de novo, X‐linked disorder, and most of them are females. Nearly all patients showed severe neurological involvement with developmental/intellectual disability. The large majority presented with epilepsy, facial dysmorphism, and brain structure and skeletal abnormalities. Less frequent was gastrointestinal, ocular, skin and other organ involvement [46, 47, 48].

On the other hand, exome sequencing of brain specimens from 56 individuals with drug‐resistant epilepsy identified five patients with somatic *de novo* SLC35A2 gene variants [49]. Since then, somatic *SLC35A2* variants have been increasingly identified in drug‐resistant focal epilepsy, early epileptic encephalopathy, and epilepsy with mild malformations of cortical development with oligodendroglial hyperplasia (MOGHE) [50, 51, 52].

#### 3.5.2 Metabolic Derangement and Genetics

The only UDP‐Gal transporter (UGT) identified in mammalian cells is SLC35A2 [53, 54]. The expression of this gene results in two splice variant products: UGT1 and UGT2, both localized in the ER and the GA [55].

SLC35A2‐CDG is an X‐linked CDG resulting from a deficiency in the UGT. This deficiency leads to a decreased galactosylation required for N‐glycan remodeling and O‐glycan synthesis within the GA, ultimately impacting the production of glycoproteins, glyco(sphingo)lipids, and proteoglycans [56]. Pedrayes et al. reported that contrary to protein glycosylation that was minimally affected, lipid glycosylation was markedly impaired, leading to the accumulation of glucosylceramide and a deficiency of digalactosylated glycosphingolipids (GSLs) and complex gangliosides [57].

Most of the more than 50 pathogenic variants reported so far are de novo; some patients had a variable percentage of mosaicism. Moreover, some variants are found in congenital and somatic phenotypes [58].

#### 3.5.3 Diagnostic Testing and Treatment

The serum Tf IEF is normal in the great majority of the patients. The exceptions with an abnormal Tf IEF show a type 2 pattern [47]. However, this can be transient [44]. Ng et al. reported in 2019 a reliable test to assess SLC35A2‐dependent UDP‐galactose transport activity in primary fibroblasts [46]. Recently serum profiling in SLC35A2‐CDG revealed hydroxylated GSLs, notably GM3, as candidate biomarkers for the disease [57].

Oral galactose supplementation improved patients' protein but also lipid glycosylation, and in most patients there was also clinical amelioration regarding growth, developmental progress, gastrointestinal symptoms, and epilepsy [57, 59].

### 3.6 SLC35A3‐CDG

#### 3.6.1 Clinical Presentation

Twelve patients have been reported, including a large kindred with eight patients and two siblings, with this defect in the uridine diphosphate N‐acetylglucosamine transporter [60, 61, 62, 63]. The clinical severity ranged from mild to profound, including death in 2 patients respectively at 21 days and 9 years. The predominant problems involved the skeleton and the joints: microcephaly, facial dysmorphism (including retromicrognathy and cleft palate), arthrogryposis (mainly of hands and feet), short long bones, abnormal vertebrae and ribs, subluxation and dislocation (shoulders, hips, knees, fingers). Neurological problems comprised mild to severe intellectual disability, epilepsy (absence epilepsy or tonic–clonic seizures), and autism spectrum disorder. Brain MRI was normal.

#### 3.6.2 Metabolic Derangement and Genetics

There was a decrease in the high‐branched glycans and an increase in lower‐branched glycans at the fibroblast cell surface. UDP‐GlcNAc transport in fibroblast‐derived Golgi vesicles was decreased. Six variants were detected: 3 missense, one nonsense, and two frameshift.

#### 3.6.3 Diagnostic Testing and Treatment

In patients with arthrogryposis, epilepsy, and intellectual disability, the UDP‐GlcNAc fibroblast transport assay is indicated or direct genetic analysis. Treatment is purely symptomatic.

### 3.7 SLC35B2‐CDG

#### 3.7.1 Clinical Presentation

SLC35B2 is a transporter of 3′‐phosphoadenosine 5′‐phosphosulfate (PAPS). Two unrelated patients have been reported with SLC35B2‐CDG (hypomyelinating leukodystrophy 26, with chondrodysplasia (HLD26)). They showed a severe intellectual disability and brain structural anomalies with hypomyelinating leukodystrophy, thin corpus callosum, and cerebral and cerebellar atrophy. Other features included pre‐ and postnatal growth retardation, dislocations of large joints, Pierre Robin sequence in one of them, chondrodysplasia, and early‐onset scoliosis [64].

#### 3.7.2 Metabolic Derangement and Genetics

Proteoglycans (PGs) are abundantly modified by the addition of sulfate to their covalently attached glycosaminoglycan (GAG) chains by membrane‐bound sulfotransferases located in the GA. The primary source of the intracellular sulfate pool comes from the extracellular environment, thanks to a specific sulfate/chloride antiporter of the plasma membrane. In the cytosol, this sulfate is then activated to the universal sulfate donor PAPS by PAPS synthases. Because most of the sulfation of glycoconjugates occurs in the Golgi apparatus, PAPS is translocated by PAPS transporters (PAPST1 and PAPST2, also named SLC35B2 and SLC35B3) from the cytoplasm into the Golgi lumen, where it serves as substrate for the sulfotransferases [65]. In vivo functional studies in both patients demonstrated that functional impairment of SLC35B2 disrupts glycosaminoglycan sulfation in fibroblasts and serum [64].

#### 3.7.3 Diagnostic Testing and Treatment

Direct gene or exome sequencing is recommended in patients with a skeletal phenotype showing hypomyelinating leukodystrophy.

Treatment is purely symptomatic.

### 3.8 SLC35C1‐CDG

#### 3.8.1 Clinical Presentation

This CDG, also called leukocyte adhesion deficiency syndrome type II (LAD II), is due to pathogenic variants in the Golgi GDP‐fucose transporter and was first reported in 1992 [66, 67]. Nineteen patients from 14 families are on record (review in [68]). Thirteen patients had a severe phenotype comprising short stature, mild facial dysmorphism, psychomotor disability, recurrent severe infections (bacterial, fungal, opportunistic) without pus, peripheral neutrophilia and the rare Bombay blood phenotype. Four patients have died at ages 1 to 4 years. Six patients showed a milder presentation with impaired speech and cognition but normal motor development, minimal immune deficiency (less severe infections, normal neutrophil counts) and no Bombay blood phenotype.

#### 3.8.2 Metabolic Derangement and Genetics

LAD II patients have a generalized deficiency of fucosylated glycoconjugates. These include the H antigen, an intermediate in the synthesis of the ABO blood group antigens, and sialyl‐Lewis X. This ligand is required for selectin‐mediated adhesion of leukocytes to vascular endothelium and subsequent extravasation to areas of infection. The presence of the Bombay blood phenotype is due to the absence of the H antigen on erythrocytes. The serum transferrin isoelectrofocusing test is, of course, normal in these patients. Twelve variants have been reported (6 missense, three nonsense, two deletions, and one splice variant). All six patients with the mild phenotype shared the p.Phe168del variant.

#### 3.8.3 Diagnostic Testing and Treatment

In the presence of recurrent severe non‐suppurative infections and a high neutrophil count, the Bombay blood group should be looked for and, if present, genetic testing performed. Oral L‐fucose supplementation showed improvement in recurrent infections and normalization of neutrophil counts in four studies [69, 70, 71, 72] but not in three other studies [73, 74, 75]. Improvement in speech, cognition and core fucosylation of serum glycoproteins was found in a patient with a mild presentation [68]. Discontinuation of fucose supplementation caused rapid loss of selectin ligands and rise of neutrophilia [76].

### 3.9 SLC37A4‐CDG

#### 3.9.1 Clinical Presentation

This CDG is due to a specific variant in the glucose‐6‐phosphate transporter 1 (G6PT1) and was first reported by Marquardt et al. in 2020. These patients present a multisystem phenotype with mild facial dysmorphism (low‐set ears, a broad nose, mandibular micrognathia and facial asymmetry), hepatopathy (increased serum transaminases and alkaline phosphatase, increased APTT, decreased fibrinogen and antithrombin, decreased factors II, V, VII and XII) and skeletal abnormalities (narrow shoulders, chest and pelvis, pectus carinatum and scoliosis). No neurological involvement was described in the reported patients [77, 78, 79, 80].

#### 3.9.2 Metabolic Derangement and Genetics

Glucose‐6‐phosphate (G6P) is normally transported from the cytosol into the ER lumen by the G6PT1. In the ER lumen it is cleaved into glucose by glucose‐6‐phosphatase‐alpha (G6Pase‐α). The resulting glucose is then exported back into the cytosol.

These patients present a dominant or de novo variant c.1267C > T (Arg423*) in SLC37A4, compromising the correct localization of the glucose‐6‐phosphate transporter due to the deletion of an ER retrieval signal. This mislocalized G6PT1 likely disrupts the critical microenvironment required for proper glycosylation [77, 81, 82].

#### 3.9.3 Diagnostic Testing and Treatment

Serum Tf IEF showed a type 2 glycosylation pattern. Mass spectrometry demonstrated that the hypoglycosylated isoforms were equally due to high mannose and hybrid glycans, a highly typical pattern distinct from those observed in other congenital disorders of glycosylation [80].

Treatment is symptomatic.

### 3.10 TMEM165‐CDG

#### 3.10.1 Clinical Presentation

Eight patients (from 6 families) have been reported with pathogenic variants in TMEM165 coding for a Golgi calcium/manganese transporter [83, 84, 85]. The predominant symptom is severe skeletal dysplasia due to important generalized demineralization (6/8). A majority of patients show growth retardation, failure to thrive, dysmorphism, hypotonia, and hepatomegaly. Less frequent are acquired microcephaly, feeding/gastrointestinal problems, and hyperthermia episodes. The following have been reported in one patient: polyhydramnios, respiratory distress, hypertonia, and nephrotic syndrome. Serum Tf IEF showed a type 2 pattern (already present in the first minutes after birth and becoming progressively more abnormal in the next 4 months) [85]. Other biochemical findings are, to varying degrees, tubular proteinuria, increased serum transaminases and creatine kinase, as well as thrombocytopenia. Four patients showed structural brain abnormalities. Two siblings died at 5 months, and another patient at 14 months.

#### 3.10.2 Metabolic Derangement and Genetics

TMEM165 (also known as SLC64A1) acts as a Ca^++^/Mn^++^:H^+^ antiporter in the medial‐ and trans‐GA network; the metal ions are pumped into the GA lumen and the protons outside. Disruption of this activity results in hypogalactosylation of four GA glycosylation pathways: protein N‐ and O‐glycosylation, glycosaminoglycan synthesis and lipid glycosylation (reviewed in [86]).

Five missense variants have been reported, as well as one splice variant.

#### 3.10.3 Diagnostic Testing and Treatment

Severe skeletal demineralization is a hallmark of this CDG. As to treatment, D‐galactose rescues the N‐glycosylation defect but not the other glycosylation defects, while Mn^++^ supplementation (e.g., as MnCl_2_) restores all GA glycosylation defects. On the other hand, D‐galactose, in combination with MnCl_2_ seems to potentiate the effect of MnCl_2_. Therefore, this combination is potentially effective in lowering the doses of manganese and hence its toxicity [83, 87].

## 4 Plasma Membrane Transporter Defect

### 4.1 SLC39A8‐CDG

#### 4.1.1 Clinical Presentation

This CDG was initially described by Park et al. [88]. Seventeen patients have been reported [89]. Pathogenic variants of *SLC39A8*, a transporter of Mn, Zn, and other divalent cations, are mainly associated with a neurological syndrome including developmental/intellectual disability, hypotonia, dystonia, dyskinesia, epilepsy, strabismus, and cerebral/cerebellar atrophy. The following are reported in a minority of patients: feeding problems, recurrent infections, skeletal changes including short stature, microcephaly, and facial dysmorphism, as well as hearing loss, mitochondrial abnormalities on muscle biopsy and, on brain MRI, abnormalities of white matter, thalamus, and basal ganglia [89].

#### 4.1.2 Metabolic Derangement and Genetics

*SLC39A8* codes for a protein known as ZIP8, a transporter crucial for zinc homeostasis, symporting a bicarbonate ion along with the metal cation. This transporter is also capable of importing other metal ions, most notably manganese (Mn^2+^), which is critical for proper glycosylation [90].

Patients with this CDG show variably decreased serum Mn and Zn levels and increased Mn and Zn in urine. Mn and Zn are cofactors for multiple enzymes including β‐1,4‐galactosyltransferase and Mn superoxide dismutase (MnSOD), causing hypoglycosylation and mitochondrial dysfunction, thus explaining the very broad clinical spectrum [91].

Ten missense variants have been reported including c.112G>C (p.Gly38Arg) in nine patients from three unrelated Hutterite families (pathogenic founder variant in this population) [89].

#### 4.1.3 Diagnostic Testing and Treatment

Serum Tf IEF was performed in 12 patients: eight showed a type 2 pattern; in two it was normal, and in two the pattern spontaneously improved. Thus, a normal pattern does not exclude this CDG. The combination of a neurological syndrome and low serum manganese should lead to exome or gene sequencing.

Oral Mn supplementation is an effective treatment option. Two patients were treated for more than 1 year with oral manganese (II)‐sulfate monohydrate. This significantly improved their motor abilities, epilepsy, and hearing, and normalized all measured enzyme deficiencies. Close monitoring of blood manganese levels is necessary to prevent manganese toxicity [92]. Less efficient is galactose supplementation that corrects the hypofunction of β‐1,4‐galactosyltransferase but not of the other manganese‐dependent metalloenzymes [92].

## 5 Discussion

This update is on CDG due to defective membrane transporters, thus excluding other cellular trafficking mechanisms such as membrane contact sites and cytoskeleton‐related pathways (broad overview in [93]). More than a century after the establishment of the membrane theory, we now understand that SLCs are essential components of cellular processes, functioning as metabolic gatekeepers. These transporters regulate the movement of substances across cell membranes and organelles, thereby maintaining cellular homeostasis. However, our knowledge of SLCs remains limited due to challenges such as substrate promiscuity, functional redundancy, and diverse tissue‐specific expression patterns [10].

Glycosylation is a critical post‐translational modification that influences protein folding, stability, and function. The GA plays a central role in this process, where various glycosylation reactions occur. Transporters, particularly those responsible for ion and metabolite transport, are essential for maintaining the optimal conditions necessary for glycosylation to proceed effectively. It is important to focus on the leading roles of these transporters in ion homeostasis, substrate availability, and the overall impact on cellular physiology.

Transporters are integral to maintaining the ionic environment within the GA, which is crucial for the activity of glycosylation enzymes. For instance, manganese (Mn^2+^) is a vital cofactor for several glycosyltransferases involved in glycan synthesis. Transporters, such as TMEM165, have been implicated in Mn^2+^ homeostasis, and their dysfunction can lead to significant glycosylation defects, as seen in TMEM165‐CDG patients. The ability of transporters to regulate ion concentrations directly affects the enzymatic activity of glycosylation pathways, underscoring their importance in ensuring that glycosylation occurs efficiently and accurately.

Moreover, Ca^2+^ ions also play a pivotal role in glycosylation processes. They are involved in the activation of certain glycosyltransferases and in the regulation of GA structure and function. Transporters that modulate Ca^2+^ levels can thus influence glycosylation outcomes. The interplay between different ions, facilitated by specific transporters, creates a fine‐tuned environment that is essential for optimal glycosylation.

In addition to ion transport, the availability of substrates for glycosylation reactions is critical. Transporters that facilitate the movement of nucleotide sugars, such as UDP‐galactose and CMP‐sialic acid, into the GA are vital for the glycosylation process. These substrates are necessary for the addition of specific sugar moieties to growing glycan chains. Any disruption in the transport of these substrates can lead to incomplete or aberrant glycosylation, which can have downstream effects on protein function and cellular signaling.

The regulation of substrate transport is also influenced by the metabolic state of the cell. For example, changes in nutrient availability can alter the expression and activity of transporters, thereby impacting glycosylation patterns. This highlights the dynamic nature of transporter functions and their integration with cellular metabolism, further emphasizing their importance in maintaining proper glycosylation.

An important SLC member of the solute carrier family is SLC35. It comprises several members of an evolutionarily conserved family of nucleotide sugar transporters (NSTs), such as the UDP‐GlcNAc (NGT) and UDP‐Gal transporters (UGT). The solute carrier family SLC35 of human NSTs is divided into seven subfamilies (SLC35A‐G), based on sequence similarity. The SLC35A subfamily includes five ancient paralogous (SLC35A1‐5), of which SLC35A1 and SLC35A3 share the highest identity with the CDG‐associated SLC35A2 [56].

SLC35D1‐CDG represents one of the most severe phenotypes among the known CDG, together with an ALG9 splice variant [94] and two ALG3‐CDG siblings with a severe malformation syndrome and intrauterine death [95].

We have not elaborated on two CDG that are possibly due to membrane transporter defects, TUSC3‐CDG and MAGT1‐CDG, because despite their known role as magnesium transporters [96], this transport function appears to be independent from their role as subunits of the oligosaccharyl transferase (OST) complex responsible for glycosylation.

Understanding the specific roles of transporters in glycosylation pathways opens avenues for potential therapeutic interventions.

Furthermore, the development of small molecules or gene therapies that enhance transporter function or compensate for deficiencies could provide new strategies for treating glycosylation disorders. As research continues to elucidate the complex interactions between transporters and glycosylation processes, it may lead to innovative approaches to manage and treat these conditions.

In summary, transporters play a fundamental role in glycosylation by regulating ion homeostasis, facilitating substrate availability, and influencing overall cellular function. They are essential in maintaining the delicate balance required for proper glycosylation. The phenotype severity when these transporters are deficient testifies to their importance (see summary of phenotypes in Table 1). Continued research into the mechanisms of transporter function and their impact on glycosylation will not only enhance our understanding of cellular biology but also pave the way for novel therapeutic strategies for glycosylation‐related disorders.

**TABLE 1:** CDG transporter defects in cells, mice and zebrafish; major findings in models and men.

	Transporters: substrate specificity	Models: cells/organisms (zebrafish and mouse)	Major findings in models	Patients: typical/frequent findings (HPO)	References: 1st report/review/update
ER transporter defects					
RFT1‐CDG	Man_5_GlcNAc_2_‐PP‐Dol flippase			Marked developmen‐tal/intellectual disability, hypotonia, seizures, hepatomegaly, coagulopathy, deafness	[12]
SLC35D1‐CDG	General UDP‐sugar transporter	Slc35d1‐deficient mice	Lethal skeletal dysplasia with severe shortening of limbs and facial dysmorphism; decreased proliferating zone with round chondrocytes and reduced proteoglycan aggregates in epiphyseal cartilage; short, and sparse chondroitin sulfate chains	Schneckenbecken‐like dysplasia, dumbbell‐shaped long bones, lateral clavicle hook, nasal hypoplasia and stillbirth up to mild phenotype into adulthood	[20, 21]
GA transporter defects					
SLC9A7‐CDG	Na+, K+/(H+) antiporter			Intellectual disability with facial dysmorphism (high anterior hairline, long face with a fine nose, deep nasolabial folds)	[24]
SLC10A7‐CDG	Putative Ca^2+^ transporter	Slc10a7−/−mouse model	Sshortened long bones; growth plate disorganization; tooth enamel anomalies; decreased heparan sulfate levels in cartilage	Amelogenesis imperfecta, skeletal dysplasia with multiple large joint dislocations, short stature and advanced bone age	[26, 27, 28, 97]
SLC26A2‐CDG	Sulfate	SLC26A2 knock‐in mouse with a partial loss of function of the sulfate transporter	Impaired sulfate uptake in chondrocytes, osteoblasts and fibroblasts	Shortening of long bones, flat epiphyses, kyphoscoliosis, advanced carpal bone age, joint contractures, abducted thumbs, delta‐shaped phalanges, hoarse voice and edema	Hästbacka et al. [98]; Forlino et al. [99]; Rapp et al. [100]; [33]
SLC35A1‐CDG	CMP‐Sia	HAP1 SLC35A1 knockout cell line with mutant (p.Gln101His) V5‐tagged SLC35A1 constructs using lentiviral transduction	Lentiviral‐mediated complementation with the disease mutation p.Gln101His failed to restore deficient O‐mannosylation in SLC35A1 knockout cells and partly restored sialylation	Neurological syndrome with macrothrombocytopenia, neutropenia and pulmonary hemorrhage	[101, 102]
SLC35A2‐CDG	UDP‐Gal, UDP‐GalNAc	Brain somatic mouse models: two Slc35a2 conditional knockout mouse models, targeting (1) the dorsal telencephalic lineage (excitatory neurons and glia) and (2) the oligodendrocytes lineage	Slc35a2 deficiency disrupts corticogenesis by delaying radial migration of neurons from the subventricular zone	Epileptic encephalopathy, cerebral visual impairment and aplasia/hypoplasia of bones of the extremities	[44, 103]
SLC35A3‐CDG	UDP‐N‐GlcNAc	Slc35a3−/−mice using CRISPR/Cas9 genome editing system	Lethal chondrodysplasia with vertebral anomalies and impaired glycosaminoglycan biosynthesis	Atypical absence status epilepticus, swan‐neck deformities of the distal phalanges, hemivertebrae and butterfly vertebrae	[60, 104]
SLC35B2‐CDG	P‐adenosine‐5′‐P‐sulfate	Zebrafish Pinscher (pic/slc35b2)	Defective sulphation of GAGs and other molecules in cartilage ultrastructure	Pre‐ and postnatal growth retardation, scoliosis, severe motor and intellectual disability and hypomyelinating leukodystrophy	[64, 105]
SLC35C1‐CDG	GDP‐Fuc	Slc35c1(−/−) mice/*Slc35c1* ^−/−^	Strongly defective leukocyte trafficking but normal lymphocyte homing to the spleen, which may explain normal lymphocyte functions in LAD II; lack of fucosylated structures; improved ricin resistance	Epilepsy, severe intellectual disability, Bombay blood group, immune deficiency and absence of pus formation at site of infection	[106, 107, 108]
SLC37A4‐CDG	Glu 6‐P (dominant)		Protein mislocalization; G6PT1 is improperly directed to the GA instead of to the ER	Hepatopathy, coagulopathy, and scoliosis	[77]
TMEM165‐CDG	Ca^2+^ and Mn^2+^	Zebrafish morpholino knockdown	Zebrafish embryos with craniofacial abnormalities, largely attributable to fewer chondrocytes	Epiphyseal, metaphyseal, and diaphyseal dysplasia	[84, 109]
Plasma membrane transporter defect					
SLC39A8‐CDG	Divalent cations: Mn, Zn, Cd, Fe	ZIP8‐iKO (Slc39a8^fl^/^fl^ UBC‐CreERT2)	Reduced expression of slc39a8 in liver, brain, kidney and small intestine; reduced levels of Mn in whole blood and tissues; defective protein N‐glycosylation; hypogalactosylation	Visual fixation instability, skeletal dysplasia with rhizomelic shortening and dwarfism	Park et al. [88]; Lin et al. [110]
		ZIP8‐LSKO (*Slc39a8* ^*fl/fl*^ *Alb‐Cre*, a liver‐specific knockout)	Decreased whole blood Mn levels; decreased Mn levels in liver, kidney, brain and heart; decreased Slc39a8 mRNA levels in liver		

## Author Contributions

D. Quelhas and J. Jaeken designed and wrote the manuscript. C. R. Ferreira provided advice and revised the manuscript. All authors have read and approved the manuscript.

## Funding

This work was partially supported by Fundação para a Ciência e a Tecnologia (FCT) to UMIB (UIDB/00215/2020 and UIDP/00215/2020) and to ITR (LA/P/0064/2020). This research was supported in part by the Eunice Kennedy Shriver National Institute of Child Health and Human Development (ZIA HD009024 to C.R.F.). The contributions of the NIH author were made as part of his official duties as NIH federal employees, are in compliance with agency policy requirements, and are considered Works of the United States Government. However, the findings and conclusions presented in this paper are those of the author and do not necessarily reflect the views of the NIH or the U.S. Department of Health and Human Services.

## Conflicts of Interest

The authors declare no conflicts of interest.

## Data Availability

Data sharing not applicable to this article as no datasets were generated or analyzed during the current study.
